# Liposomal Fba and Met6 peptide vaccination protects mice from disseminated candidiasis

**DOI:** 10.1128/msphere.00189-24

**Published:** 2024-06-21

**Authors:** Wei-Chiao Huang, Karen Eberle, Jonothan Rosario Colon, Jonathan F. Lovell, Hong Xin

**Affiliations:** 1 Department of Biomedical Engineering, State University of New York at Buffalo, Buffalo, New York, USA; 2 Department of Microbiology, Immunology & Parasitology, LSU Health Sciences Center New Orleans, New Orleans, Louisiana, USA; Yonsei University, Seoul, South Korea

**Keywords:** vaccines, candidiasis, spontaneous nanoliposome antigen particle

## Abstract

Epitopes from the *Candida* cell surface proteins Fba and Met6 are putative vaccine targets for invasive candidiasis. Here, we describe a *Candida* vaccine approach in which short peptides derived from Fba and Met6 are used in spontaneous nanoliposome antigen particle (SNAP) format. SNAP was enabled by the interaction of cobalt porphyrin phospholipid in liposomes with three histidine residues on the N-terminus of synthetic short peptide immunogens from Fba (F-SNAP), Met6 (M-SNAP), or bivalent Fba and Met6 (FM-SNAP). Liposomes were adjuvanted with synthetic monophosphoryl lipid and QS-21. In mice, immunization with F-SNAP, M-SNAP, or FM-SNAP induced antigen-specific IgG responses and mixed Th1/Th2 immunity. The duplex FM-SNAP vaccine elicited stronger antibody responses against each peptide, even at order-of-magnitude lower peptide dosing than a comparable adjuvanted, conjugate vaccine. Enzyme-linked immunosorbent spot analysis revealed the induction of antigen-specific, cytokine-producing T cells. Compared to F-SNAP or M-SNAP, higher production of TNFα, IL-2, and IFNγ was observed with re-stimulation of splenocytes from bivalent FM-SNAP-immunized mice. When vaccinated BALB/c mice were challenged with *Candida auris*, analysis of the fungal burden in the kidneys showed that SNAP vaccination protected from disseminated candidiasis. In a lethal fungal exposure model in A/J mice, F-SNAP, M-SNAP, and FM-SNAP vaccination protected mice from candidiasis challenge. Together, these results show that further investigation into the SNAP adjuvant platform is warranted using Fba and Met6 epitopes for a pan-*Candida* peptide vaccine that provides multifaceted protective immune responses.

**IMPORTANCE:**

This study introduces a promising vaccine strategy against invasive candidiasis, a severe fungal infection, by targeting specific peptides on the surface of *Candida*. Using a novel approach called spontaneous nanoliposome antigen particle (SNAP), we combined peptides from two key *Candida* proteins, Fba and Met6, into a vaccine. This vaccine induced robust immune responses in mice, including the production of protective antibodies and the activation of immune cells. Importantly, mice vaccinated with SNAP were shielded from disseminated candidiasis in experiments. These findings highlight a potential avenue for developing a broad-spectrum vaccine against *Candida* infections, which could significantly improve outcomes for patients at risk of these often deadly fungal diseases.

## INTRODUCTION

Fungal infections exert a significant burden on society and account for ~1.5 million deaths worldwide annually. In 2022, the World Health Organization reported the first ever fungal priority pathogens list, classifying *Candida auris* and *Candida albicans* in the highest priority group (1). Candidiasis ranks third of all nosocomial bloodstream infections, and despite proper antifungal therapy, at least 40%–50% of affected individuals will die (2, 3). In particular, *C. auris* is a multidrug-resistant health care-associated fungal pathogen that has emerged as the first fungal pathogen to be a global public health threat. *C. auris* infection occurs as community-acquired infections as well as infections in the hospital setting, where isolates show multidrug resistance and high mortality (4–7). During the COVID-19 pandemic, intensive care units (ICUs) filled to capacity, contributing to numerous *C. auris* ICU outbreaks globally (8–10). Despite the substantial global burden of human fungal infections, there are no approved fungal vaccines to protect at-risk individuals. Previous studies have shown that synthetic peptide and glycopeptide *Candida* immunogens and subsequently a chimeric double-peptide vaccine against *C. albicans* cell surface epitopes induced antibody-mediated protection in mice against disseminated candidiasis (11–16). We developed fully synthetic peptide vaccines using cell surface epitopes derived from *C. albicans* cell wall proteins, fructose bisphosphate aldolase (Fba) and methionine synthase (Met6), which are expressed during human invasive candidiasis (17, 18). The two conserved peptides, Fba and Met6, both have strong identity among all five medically relevant *Candida* species causing ~99% of human candidiasis, including *C. auris* (with the Fba peptide showing 80% sequence identity and Met6 showing 100% identity). Hence, these epitopes could make up a “universal” vaccine candidate to protect against all medically relevant, invasive *Candida* infections. We have also demonstrated that vaccine-elicited antibodies can be passively transferred and antibody-mediated protection against candidiasis can be achieved in both immunocompetent and neutropenic mice (19).

One challenge and consideration for a pan-*Candida* vaccine is that serious invasive *Candida* infections usually impact immunocompromised individuals who may not mount optimal responses. Therefore, potent vaccine delivery systems and vaccine adjuvants are likely to play a role. To continue our efforts in advancing an effective antifungal peptide vaccine, we herein assess a spontaneous nanoliposome antigen particle (SNAP) vaccine system with the previously validated Fba and Met6 synthetic peptide immunogens. SNAP is a liposomal vaccine platform based on the inclusion of cobalt-porphyrin-phospholipid (CoPoP) within a phospholipid bilayer (20). CoPoP interacts with polyhistidine sequences, effectively anchoring them into the bilayer (21). As such, it is a method for biostable antigen display on a nanoscale all-lipid scaffold (22). Additional vaccine adjuvants including monophosphoryl lipid A (MPLA) and QS-21 are included, as these have been shown to be effective human adjuvants (23). SNAP has been shown to induce potent antibody responses (24) and cellular responses (25) and is also amenable to multivalent protein or peptide immunization (26, 27). More recently, SNAP was translated into clinical testing, where it safely and effectively induced durable antibody responses in a COVID-19 vaccine in phase 2 trials (28, 29). In the present study, mouse models of disseminated candidiasis are used to determine the efficacy of SNAP vaccines using the Fba and Met6 immunogens, which are modified with three histidine residues to enable liposome display based on CoPoP interaction.

## MATERIALS AND METHODS

### *Candida* isolates and culture conditions

*C. auris* clinical isolate AR-0389 (CAU-09) was supplied by the U.S. Centers for Disease Control and Prevention (CDC; Atlanta, GA, USA). *C. albicans* reference strain SC5314 was supplied by the American Type Culture Collection (MYA-2876, ATCC, Manassas, VA, USA). Yeast cells were grown overnight in 25 mL glucose yeast extract peptone (GYEP) broth in an orbital shaker at 37°C. For all *in vitro* assays, the inocula were serially passaged daily for 3 days to allow yeast to reach the stationary phase and then washed three times in Dulbecco’s phosphate-buffered saline (DPBS). Cell density was measured using a hemocytometer and adjusted to the desired density in DPBS.

### Mice

Female A/J mice were purchased from Jackson Laboratory, and female ICR mice were purchased from Envigo. Female BALB/c mice were purchased from Jackson Laboratory. Few studies have reported known differences in immune responses between male and female mice, with respect to *C. auris* invasive infection. To minimize variables in these initial SNAP studies, we focused solely on female mice; however, future studies should include both sexes.

### Materials

The following lipids were used to form CoPOP/PHAD/QS21 liposomes (CPQ): 1,2-dipalmitoyl-sn-glycero-3-phosphocholine (Corden Cat # LP-R4-070), cholesterol (PhytoChol, Wilshire Technologies), synthetic monophosphoryl hexa-acyl lipid A, 3-deacyl (PHAD-3D6A, Avanti Cat # 699 855), and QS-21 (Desert King). Cobalt-porphyrin-phospholipid and porphyrin-phospholipid were produced as previously described (30). Peptides were synthesized by GenScript. The sequence used for Fba was HHH-GVIYGKDVKDLFDYAQE and HHH-GFPRIGGQRELKKITEA for Met6.

### Liposome preparation

CoPoP, PHAD-3D6A, and QS-21-containing liposomes (CPQ) were prepared by an ethanol injection method, followed by nitrogen-pressurized lipid extrusion in a phosphate-buffered saline (PBS) conducted at 55°C. The remaining ethanol was removed by dialysis against PBS twice at 4°C. The final liposome concentration was adjusted to 320 µg mL^−1^ of CoPoP, 128 µg mL^−1^ of PHAD, and 128 µg mL^-1^ of QS21, and liposomes were passed through a 0.2 µm sterile filter and stored at 4°C. The liposome size and polydispersity index (PDI) were determined by dynamic light scattering with a NanoBrook 90 plus PALS instrument, after 200-fold dilution in PBS.

### Peptide-SNAP preparation

The vaccines were prepared by incubating the peptide at a concentration of 80 µg mL^−1^ with liposomes (CoPoP or equivalent concentration of 320 µg mL^−1^) for 3 h at room temperature followed by 4°C incubation overnight prior to dilution for immunization.

### Murine immunization and serum analysis

Five- to six-week-old female mice received intramuscular (I.M.) immunizations on days 0 and 21 containing 1.5 µg peptide (Fab or Met6) combined with CPQ liposomes (6 µg CoPoP, 2.4 µg QS21, and 2.4 µg PHAD). For bivalent vaccine formulation, mice received 1.5 µg of Fab peptide/1.5 µg of Met6 peptide combined with CPQ liposomes (12 µg CoPoP, 4.8 µg QS21, and 4.8 µg PHAD). The vaccines were formulated in 50 µL and injected into the caudal thigh muscle for the I.M. route. The final bleeding was collected on day 42.

### ELISA assay

Anti-Fba, Anti-Met6, and anti-His-tag IgG titers were assessed by ELISA in 96-well plates, coated with 100 µL of peptide (Fab, Met6, or His-tag) (GenScript Cat # RP11737), diluted in coating buffer (3.03 g Na_2_CO_3_; 6 g NaHCO_3_ in 1 L distilled water, pH 9.6) to 2.5 µg/mL. After 2 h at 37°C, the plates were washed and then blocked with 2% BSA in PBS containing 0.1% Tween-20 (PBS-T) for an additional 2 h at 37°C. Murine sera (diluted 1:100 in PBS-T containing 1% BSA, 100 µL per well) were added to the top well and diluted 1:100 to 1:106 down the plate. They were incubated for 1 h at 37°C, followed by washing with PBS-T. Anti-mouse IgG-HRP (Cell Signaling Technology, Cat. # 7076S) was added, followed by incubation for 30 min at 37°C. Wells were washed again with PBS-T before the addition of tetramethylbenzidine solution. Titers were defined as the reciprocal serum dilution at which the absorbance at 450 nm exceeded the background by greater than one absorbance unit.

### *In vivo* model of disseminated infection

All groups were challenged after the final booster with either lethal dose of live *C. auris* AR-0389 2 × 10^8^ or *C. albicans* SC5314 2 × 10^5^ intravenously, and the experiment was terminated on either day 21 or day 28 post-challenge. Five mice from each group were sacrificed 72 h after the challenge for fungal burden analysis in the kidney. All mice were monitored daily for death or the development of a moribund state, at which point they were sacrificed via CO_2_ inhalation. All surviving mice were sacrificed at the conclusion of each study.

### Quantification of fungal burdens

The pair of kidneys were extracted from mice, and each organ was homogenized in DPBS. The homogenate was then serial diluted and plated onto GYEP agar plates containing chloramphenicol. The plates were incubated for 48 h at 37°C at which time CFUs were quantified. The limit of detection was 50 CFUs/g per organ.

### Enzyme-linked immunosorbent spot assay

Splenocytes were harvested from immunized BALB/c mice on day 42. Spleens were collected and passed through a 70 µm cell strainer in a 50 mL tube to collect single cells. The cells were centrifuged at 500 rcf, and a red blood lysis buffer was added for 5 min on ice to lyse the red blood cells. After incubation, 30 mL of PBS was added to dilute the lysis buffer, and the samples were centrifuged at 500 rcf for 5 min. A total of 3 × 105 splenocytes were seeded in pre-coated murine IFN-γ/IL-2 or murine IFN-γ/TNFα enzyme-linked immunosorbent spot (ELISpot) plate, and 10 µg/mL of Fab peptide or Met6 peptide was added to each well. The cells were cultured in 5% CO_2_/95% air at 37°C in a humidified chamber for 24 h. The detection of spots was performed according to the manufacturer’s instructions from Immunospot, using the Murine IFN-γ/IL-2 Double-Color ELISPOT kit or Murine IFN-γ/TNFα Double-Color ELISPOT kit. The next day, the plate was washed twice with PBS and twice with PBS-T. The wells were incubated with 80 µL of anti-murine IFN-γ/IL-2 detection buffer or anti-murine IFN-γ/ TNFα detection buffer for 2 h at RT. Later, the wells were washed three times with PBS-T, and then incubated with streptavidin-AP for 1 h. After the incubation, each well was washed with PBS-T twice and distilled water twice. To develop the blue and red spots, the plates were incubated for 15 min at RT with 80 µL per well of blue developer solution provided by the manufacturer, followed by rinsing the wells with tap water three times to stop the reaction. Then, the red developer solution was added and incubated for 5 min, and the reaction was stopped by rinsing the wells with tap water three times. The images were acquired with a CTL ImmunoSpot S6 FluoroCore analyzer.

### Statistical analysis

Data were analyzed by GraphPad Prism 9 software (GraphPad, Inc.). ELISA data were assessed for statistical significance by curve fit analysis. Survival times were statistically evaluated by Kaplan–Meier (GraphPad Prism, version 9). Data from CFU counts were analyzed by two-tailed Student’s *t* test. Multiple comparisons were made by analysis of variance (one-way ANOVA) followed by Newman-Keuls post-test. *P* values < 0.05 were considered significant.

## RESULTS

### Liposome-displayed peptide vaccines generate high titer of antigen-specific IgG

**Fig 1:**
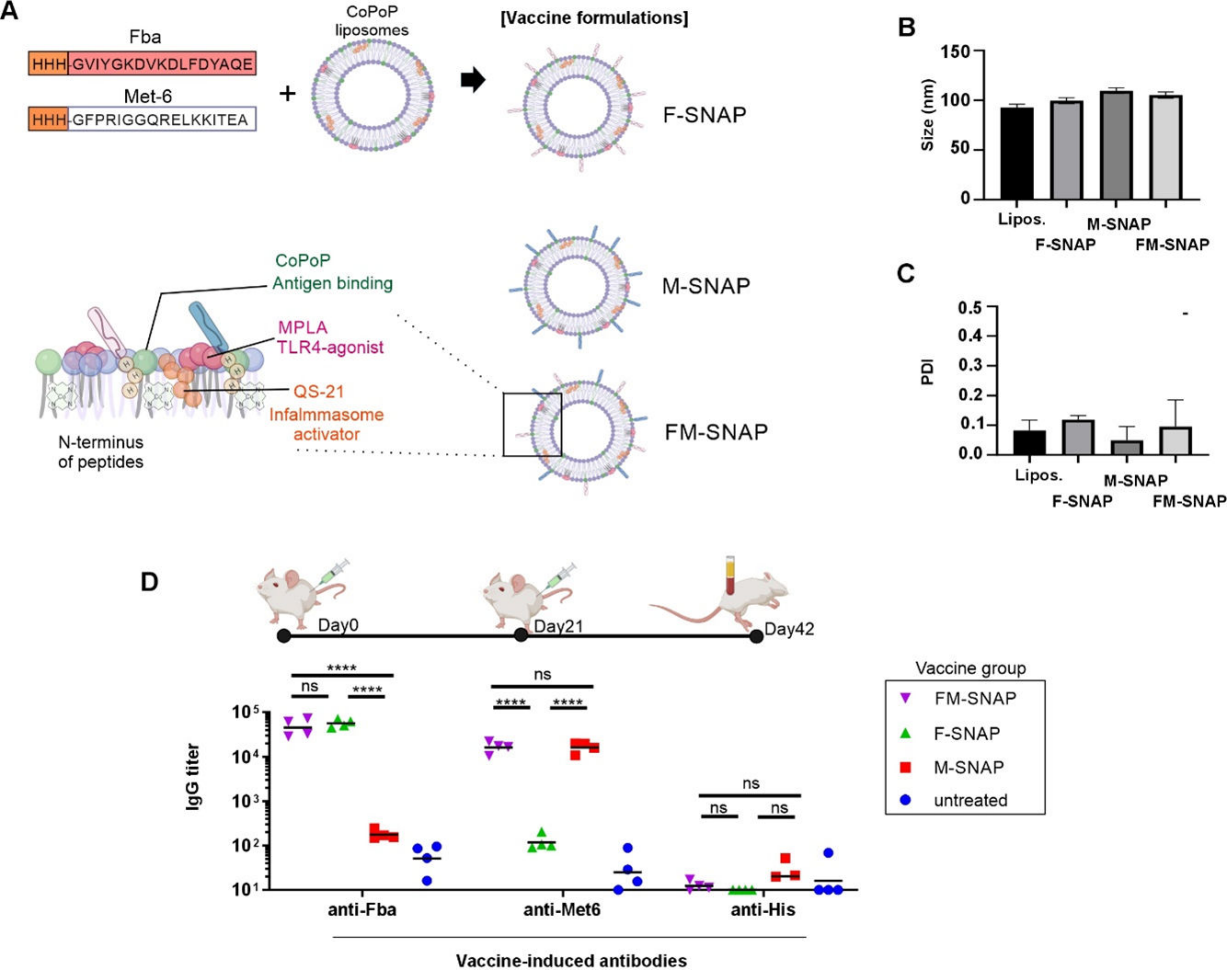
SNAP vaccines induce antigen-specific anti-Fba and anti-Met6 IgG responses. (**A**) Schematic representation of spontaneous nanoliposome antigen particles. Fba or Met-6 peptides that are modified with three histidine residues and mixed with liposomes that include cobalt porphyrin phospholipid. Interaction of the abbreviated his tag and intrabilayer cobalt results in a stable display of peptides on liposome surfaces. In addition, the immunostimulatory adjuvants MPLA and QS21 are incorporated into the bilayer. (**B**) Particle size and (**C**) PDI before and after peptide binding. (**D**) Outbred ICR mice were immunized on days 0 and 21 with F-SNAP, M-SNAP, or FM-SNAP (1.5 µg per peptide), and sera were collected on day 42 for ELISA analysis of antigen-specific IgG as indicated.

The concept of Fba and Met6 peptide display using SNAP is shown in Fig. 1A. Upon admixture, the peptides insert into the bilayer of liposomes containing CoPoP. Three vaccines were assessed: F-SNAP (displaying Fba peptide), M-SNAP (displaying Met6 peptide), and FM-SNAP (co-displaying Fba and Met6 peptides). As shown in Fig. 1B and C, the size and polydispersity index of the liposomes were measured with or without incubation with the antigens. The particle size did not change substantially and remained close to 100 nm, while the PDI remained low, reflecting a monodisperse population. Outbred CD-1 mice, which represent a more diverse population than inbred mice, were immunized intramuscularly with the three vaccine types. As shown in Fig. 1D, F-SNAP induced Fab antigen-specific IgG, M-SNAP elicited Met6 antigen-specific IgG, and FM-SNAP induced both. No significant anti-hexa his tag antibodies were observed, which is not surprising considering that the truncated his-tag on the antigens was only three histidine residues.

### Protective immunity induced by SNAP vaccines in BALB/c mice

**Fig 2:**
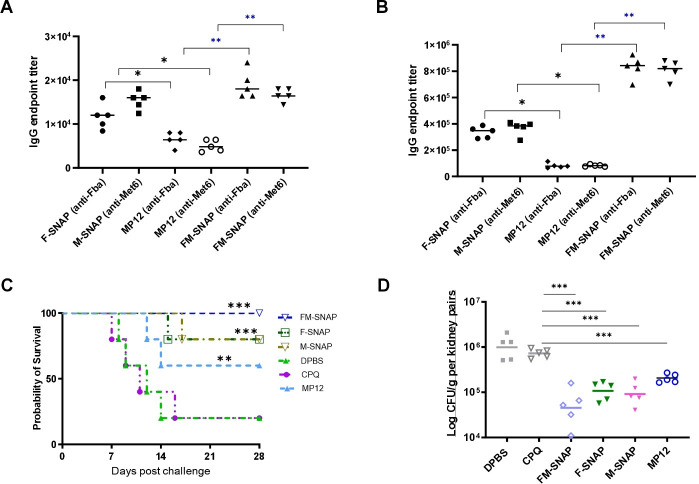
Peptide-SNAP vaccines elicit enhanced anti-Fba and anti-Met6 IgG responses at lower vaccine dosages compared to our previously optimized MP12 vaccine comparator. Peptide-specific IgG endpoint titers were evaluated via ELISA after primary injection (**A**) and first booster of immunization (**B**) in BALB/c mice, *n* = 5. Peptide-SNAP (1.5 µg); MP12 (15 µg) was used as a positive control for IgG endpoint titers. Each dot represents an individual animal, and the bars depict the median. Differences between vaccinated groups were analyzed by K-W test (one-way ANOVA) using the MP12 group as a reference. Prism 9 software was used for all statistical analyses. *P < 0.01 and **P < 0.001. (**C**) All groups were challenged after the final booster with a lethal dose of live *C. auris* AR-0389 2 × 10^8^ intravenously, and the experiment was terminated on day 28 post-challenge. Survival times were statistically evaluated by log-rank (Mantel-Cox) test. **P < 0.01 and ***P < 0.001. (**D**) BALB/c mice (*n* = 5) were sacrificed 72 h after the challenge for fungal burden analysis in the targeted organ kidney. Groups were analyzed by one-way ANOVA followed by Tukey’s test using log-transformed data, immunized groups had significantly reduced CFUs (*P < 0.05*,* **P < 0.005*,* ***P < 0.0005*,* and ****P < 0.0001) as compared to adjuvant (CPQ)-alone group.

Encouraged by the initial SNAP immunogenicity results, we next assessed the peptide vaccine in inbred BABL/c mice, which are commonly used for preclinical candidiasis models. We also made use of a previously reported comparator vaccine, MP12, which is a Fba-Met6 conjugate also including a tetanus toxin epitope and an integrin ligand, formulated with Adjuplex to enhance the efficacy against disseminated candidiasis (16). We first compared peptide-specific IgG responses induced by peptide-SNAP to that of MP12 following the same I.M. immunization route and schedule. In BALB/c mice, 1.5 µg of F-SNAP, M-SNAP, or FM-SNAP all induced more robust IgG responses with a 10-fold less antigen dose after primary immunization (Fig. 2A) and boosting (Fig. 2B) as compared to MP12 (15 µg). Next, mice were challenged intravenously with *C. auris*. SNAP peptide vaccines induced good protection, as evidenced by the significantly prolonged survival observed in the vaccinated BALB/c groups (Fig. 2C). Five mice of each group were sacrificed 72 h after challenge for fungal burden analysis in the kidney, the targeted organ. Importantly, SNAP-vaccinated groups had significantly reduced kidney CFU comparable to MP12 (Fig. 2D).

### Protective immunity induced by SNAP vaccines in A/J mice

**Fig 3:**
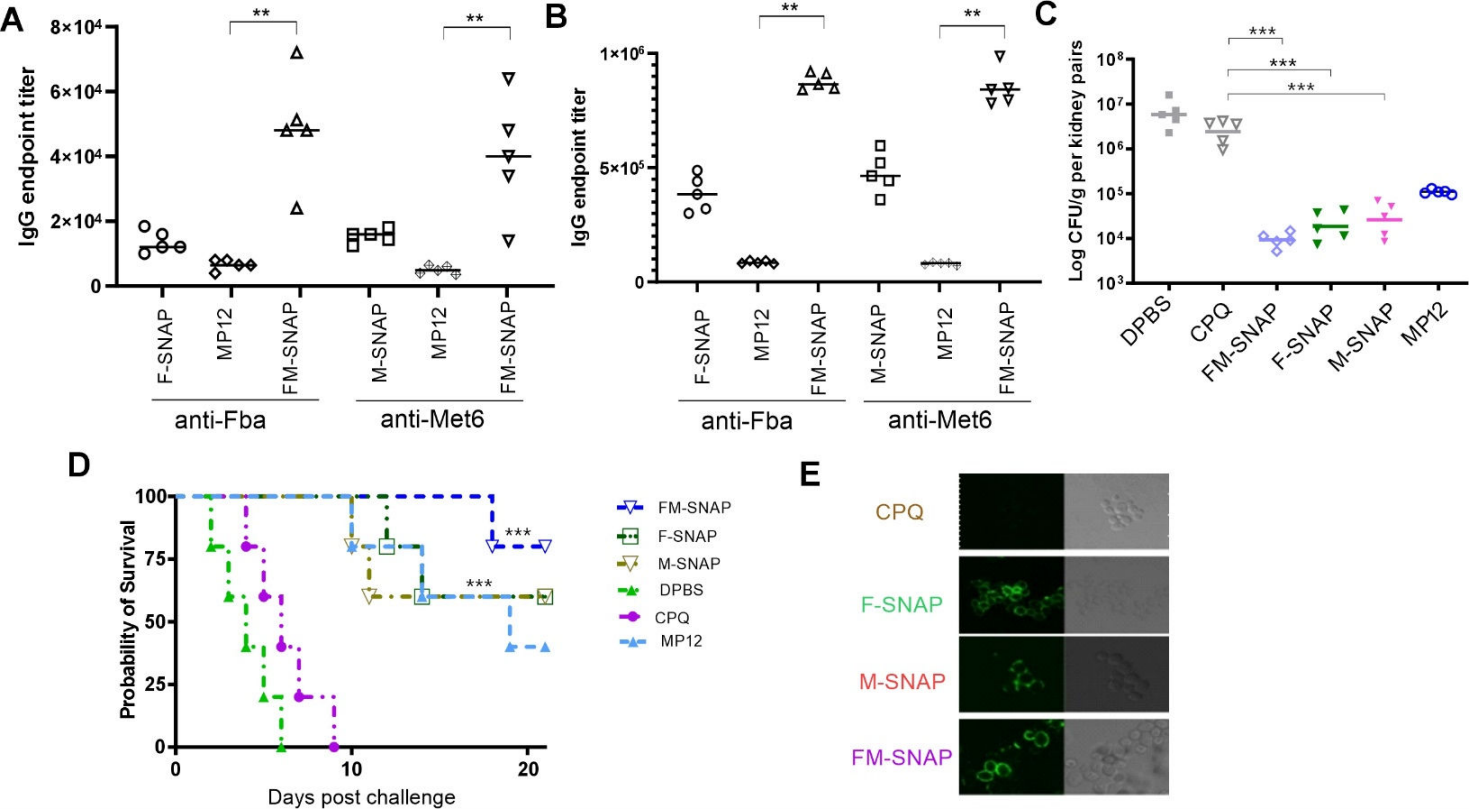
SNAP vaccines induce protective immunity in A/J mice. A/J mice (female, 7 weeks and *n* = 10) were I.M. vaccinated and boosted with peptide-SNAP (1.5 µg) or MP12 (15 µg) in 2-week intervals. Peptide-specific IgG endpoint titers were evaluated via ELISA after primary injection (**A**) and first booster of immunization (**B**) in mice, *n* = 5. Peptide-SNAP (1.5 µg); MP12 (15 µg) was used as a positive control for IgG endpoint titers. Each dot represents an individual animal, and the bars depict the median. Differences between vaccinated groups were analyzed by K-W test (one-way ANOVA) using the MP12 group as a reference. Prism 9 software was used for all statistical analyses. *P < 0.01 and **P < 0.001. All groups of A/J mice were then challenged i.v. with a lethal dose of live *C. auris* AR-0389 2 × 10^8^ CFU 14 days after final immunization. (**C**) Five mice of each group were sacrificed 72 h after the challenge for fungal burden analysis in the kidney. Immunized groups had reduced CFUs (***P < 0.0001) compared to the adjuvant (CPQ)-alone group. Fungal burden in organs as determined by CFU counts per gram was analyzed with Mann-Whitney’s non-parametric test when two groups were compared. ***P < 0.001. (**D**) Twenty-one-day survival curve from 7‐week‐old female A/J mice (*n* = 5) vaccinated with peptide-SNAP (1.5 µg) or MP12 (15 µg). DPBS and CPQ (adjuvant alone) were used as controls. Survival was evaluated by the log-rank (Mantel-Cox) test. **P < 0.01 and ***P < 0.001. (**E**) The binding of functional antibodies in immune sera from vaccinated A/J mice to live *C. auris* AR-0389 yeast cells was determined by immunofluorescence. The serum sample from the adjuvant-alone (CPQ) group (negative control), (F-SNAP, M-SNAP, and FM-SNAP): immune sera from F-SNAP-, M-SNAP-, and FM-SNAP-vaccinated groups. Serum dilution 1:100, Alexa Fluor 488‐conjugated goat anti‐mouse IgG as secondary antibody.

Our prior study demonstrated that MP12 protects both immunocompetent C57BL/6 and IL-17RA k/o mice from lethal *C. albicans* challenge, and the mixed IgG2a/IgG1 immunity played a key role (16). We additionally validated the peptide-SNAP vaccine in highly susceptible A/J mice, which exhibit different MHC haplotypes. Notably, we established a susceptible A/J mouse model (C5 deficiency) for systemic *C. auris* infection (31, 32). Employing the same I.M. immunization route, dosage, and schedule, we compared the peptide-specific IgG responses induced by peptide-SNAP to those elicited by MP12 in A/J mice. Consistent with the findings in BALB/c mice, the peptide-SNAP vaccines induced significantly higher levels of peptide-specific IgGs with a 10-fold lower dose after both primary immunization (Fig. 3A) and the first booster (Fig. 3B), as compared to our prototype vaccine MP12. Vaccinated groups also had significantly prolonged survival (Fig. 3D) by day 28 post-infection and significantly reduced kidney CFU at 72-h timepoint post-lethal challenge (Fig. 3C). SNAP vaccines demonstrate superior protection compared to MP12, as supported by the data showing higher rates of survival and reduced fungal burden. Peptide-SNAP-induced specific antibodies in immune sera were able to bind to live *C. auris* AR-0389 cells (Fig. 3E).

### The SNAP platform induced mixed Th1/Th2 immunity

**Fig 4:**
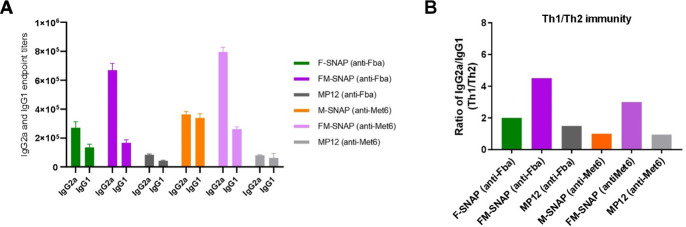
SNAP vaccines induce anti-Fba and anti-Met6 IgG2a and IgG1 responses reflecting mixed Th1/Th2 immunity. (**A**) BALB/c mice (*n* = 5) were intramuscularly vaccinated and boosted twice in 2-week intervals with peptide-SNAP, and MP12 was used as a control. Sera were drawn, and endpoints of IgG2a and IgG1 subclass titers were measured by ELISA 2 weeks after final boosting. (**B**) Th1-biased mixed Th1/Th2 (IgG2a/IgG1) responses were induced by peptide-SNAP in vaccinated mice.

Our prior study demonstrates that MP12 in Adjuplex protects both immunocompetent C57BL/6 and IL-17RA k/o mice from lethal *C. albicans* challenge, and the mixed Th1/Th2 responses shown by IgG2a/IgG1 ratio played a key role (16). MP12-vaccinated mice also demonstrated significantly prolonged survival when challenged 5 months after the final booster, indicating the induced long-term immunological memory of protective immunity (16). Similarly, peptide SNAP vaccines induced robust specific IgG2a and IgG1 responses in vaccinated BALB/c mice (Fig. 4A) and endpoint titers of IgG2a/IgG1, indicating mixed Th1/Th2 immunity (Fig. 4B). After the lethal challenge, all vaccinated mice had significantly reduced kidney CFU as compared to controls (Fig. 2C), and FM-SNAP group had the least CFU, indicating SNAP adjuvant platform functions well to set an immune environment that supports both humoral and cellular mechanisms crucial to protection against invasive candidiasis.

### SNAP vaccines protected BALB/c mice against disseminated *C. albicans* infection

**Fig 5:**
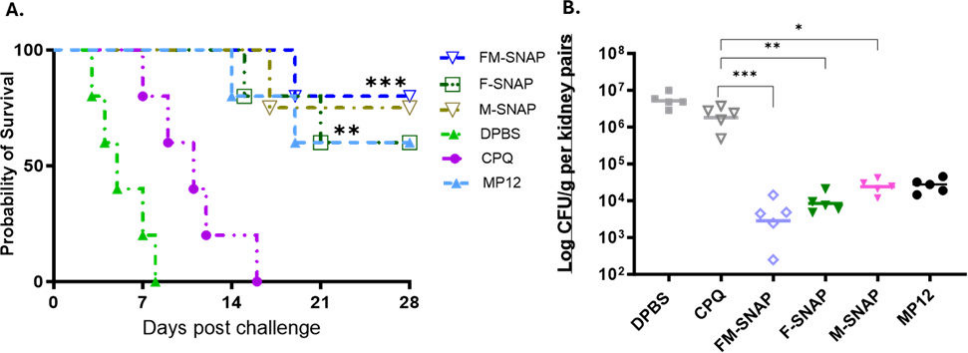
Peptide-SNAP vaccines protected BALB/c mice against disseminated candidiasis caused by *C. albicans* SC5314. Antigen-specific IgG endpoint titers were evaluated via ELISA after prime and boost immunization in BALB/c mice, *n* = 10. All groups were challenged 14 days after the final booster with a lethal dose of live *C. albicans* SC5314 2 × 10^5^ intravenously, and the experiment was terminated on day 28 post-challenge. (**A**) Survival times were statistically evaluated by log-rank (Mantel-Cox) test. **P < 0.01 and ***P < 0.001. (**B**) BALB/c mice (*n* = 5) were sacrificed 72 h after the challenge for fungal burden analysis in the targeted organ kidney. Groups were analyzed by one-way ANOVA followed by Tukey’s test using log-transformed data, and immunized groups had significantly reduced CFUs (*P < 0.05*,* **P < 0.005*,* and ***P < 0.0005) as compared to adjuvant (CPQ)-alone group.

We repeated the same immunizations in BALB/c mice and obtained comparable results: peptide-SNAP vaccines induced much higher levels of peptide-specific IgGs following each immunization. Furthermore, we validated the protective efficacy of peptide-SNAP vaccines in the BABL/c mouse model of invasive *C. albicans* infection. Following the same I.M. immunization route, dosage, and schedule, all groups were challenged 14 days after the final booster with a lethal dose of live *C. albicans* SC5314 2 × 10^5^ intravenously, and the experiment was terminated on day 28 post-challenge. Vaccinated groups had significantly prolonged survival (Fig. 5A) by day 28 post-infection and significantly reduced kidney CFU at 72-h time point post-lethal challenge (Fig. 5B). SNAP vaccines demonstrated improved protection compared to MP12, as supported by the data showing higher rates of survival and reduced fungal burdens.

### SNAP vaccines induce antigen-specific T cells that secrete IL-2, IFN-γ, and TNF-α

**Fig 6:**
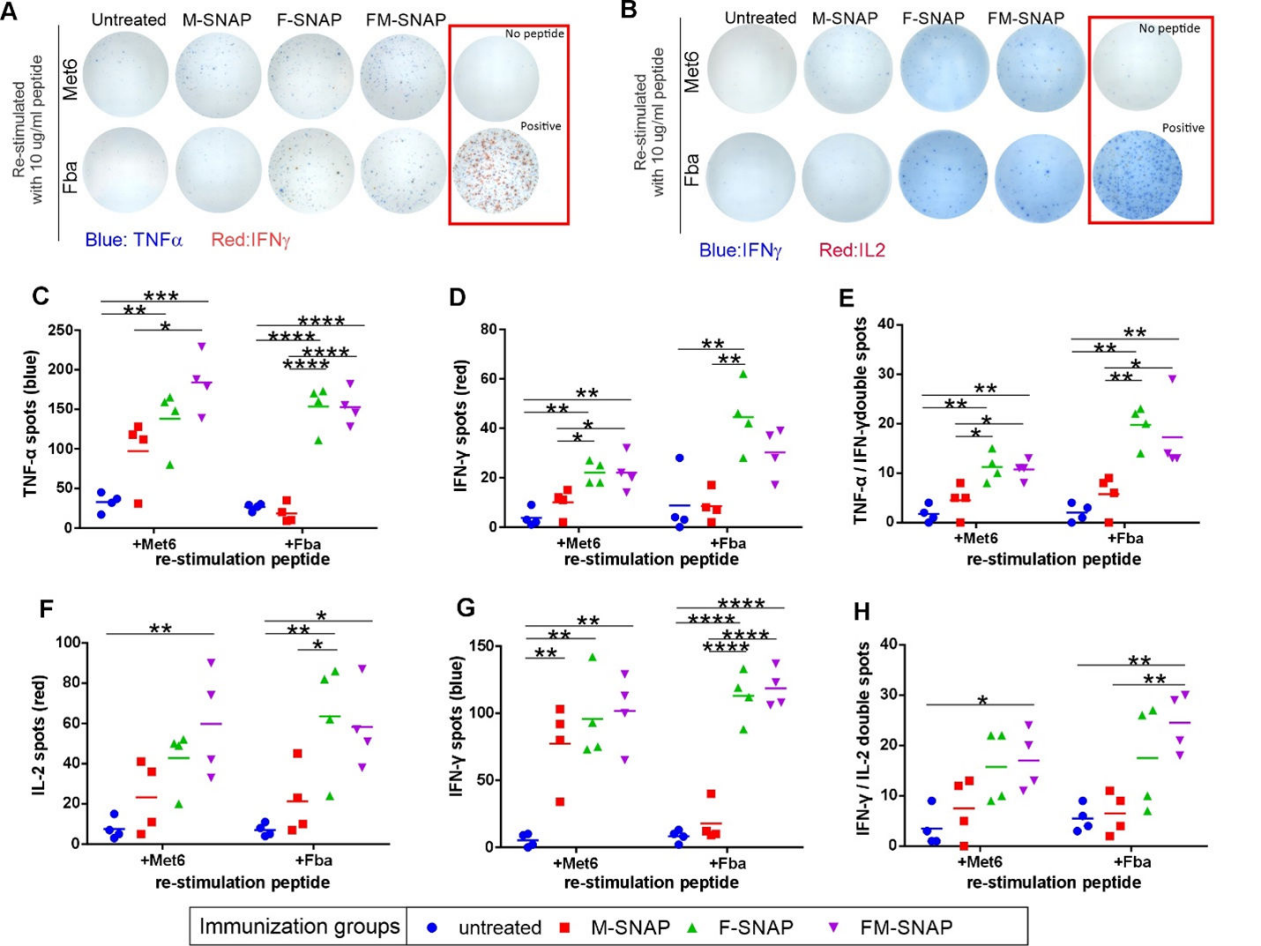
Antigen-specific cellular responses induced by FM-SNAP immunization. BALB/c mice were immunized with peptide-SNAP on days 0 and 14. Splenocytes were collected and restimulated with peptides (Met6 or Fba). Images of ELISpot results formulating (**A**) blue spots representing TNFα or red spots representing IFNγ or (**B**) blue spots representing IFNγ or red spots representing IL2 from spleen stimulated with either Fba or Met6 peptide. Quantification of results is shown for (**C**) TNFα, (**D**) IFNγ, and (**E**) TNFα/IFNγ overlap spots or (**F**) IL2, (**G**) IFNγ, and (**H**) IL2/IFNγ overlap spots. The lines in panels **C–H** represent mean and one-way ANOVA followed by Tukey’s test to analyze the differences, *P < 0.05, **P < 0.01, ***P < 0.001, and ****P < 0.00001. Spleen cells from *n* = 4 mice per group.

Next, we conducted two ELISpot assays, TNFα/IFNγ ELISpot and IFNγ/IL2 ELISpot assays, to detect antigen-specific T cells after post-immune spleen were re-stimulated with Met6 and Fba peptide. As shown in Fig. 6A, after re-stimulating with Met6 or Fba peptide, F-SNAP- and FM-SNAP-immunized BALB/c mice developed higher specific TNFα and IFNγ-producing cells. The number of peptide-specific spot-forming cells (SFC) was assessed in the spleen that developed higher specific TNFα-producing cells (Fig. 6C), IFNγ-producing cells (Fig. 6D), and specific TNFα/IFNγ-producing cells (Fig. 6E). A similar trend is shown in Fig. 6B, after re-stimulated with Met6 or Fba peptide, F-SNAP and FM-SNAP-immunized mice developed higher specific IL2- and IFNγ-producing cells. The number of peptide-specific SFC was assessed in the spleen that developed higher specific TNFα-producing cells (Fig. 6F), IFNγ-producing cells (Fig. 6G), and specific TNFα/IFNγ-producing cells (Fig. 6H). The data provide valuable information about the functional characteristics of the immune responses induced by FM-SNAP and the presence of antigen-specific T cells capable of secreting multiple cytokines simultaneously with antigen restimulation, which should be beneficial for effective immune responses against *Candida*. Generally, the non-immunized control mice did not induce cytokines upon antigen restimulation, whereas the FM-SNAP groups produced the highest responses. In some cases, the M-SNAP group produced cytokines upon restimulation with the Fba, although levels were low. Further work is required to elicit the nature and antigen specificity of the T-cell responses, including the phenotype of antigen-specific T cells.

## DISCUSSION

Short peptides, like the Fba and Met6 fragments used here, typically have poor immunogenicity on their own due to their hapten-like nature. Our approach toward a synthetic anti-*Candida* vaccine has gone through multiple iterations, progressing from glycopeptide conjugates to double peptide constructs. The initial two generations of the vaccine provided substantial protection in mice; however, they necessitated dendritic cells as a delivery platform, which is costly, labor-intensive, and impractical for human use. Our third iteration of the anti-*Candida* peptide vaccine, MP12, comprises a Fba-Met6 conjugate containing various amino acid spacers and T-cell universal epitopes aimed at enhancing immunogenicity and stability. When formulated with Adjuplex, MP12 demonstrated protection against disseminated candidiasis in both immunocompetent and immunocompromised mouse models without relying on dendritic cells as a delivery platform (16). In this study, we introduced the fourth iteration of the anti-*Candida* peptide vaccine, the FM-SNAP vaccine, which utilizes a double-peptide format. This format offers several advantages compared to single-peptide vaccines like Hyr1 (33) or Als3 (34). By targeting multiple conserved epitopes, our vaccine aims to broaden the immune response and enhance protection against *Candida* infections. We are considering conducting comparative studies to evaluate the efficacy of our FM-SNAP vaccine in comparison with other anti-fungal vaccines, including Hyr1 and Als3, to assess their relative effectiveness and immunogenicity.

The SNAP vaccine adjuvant system technology has recently been shown to be safe and effective in phase 2 clinical trials (28, 29) for a COVID-19 vaccine. This study demonstrated SNAP is a potent and effective adjuvant platform for an anti-*Candida* peptide vaccine for both Fba and Met6. F-SNAP, M-SNAP, and FM-SNAP each elicited significantly stronger IgG responses, requiring over a 10-fold lower antigen dose after the first booster compared to MP12 (15 µg) formulated with Adjuplex in ICR (CD-1) outbred mice. We replicated this immunization in inbred BALB/c mice, and all three peptide-SNAP vaccines significantly enhanced the immunogenicity of the Fba/Met6 peptide, generating elevated levels of peptide-specific IgG responses and rapid immune memory after a single primary immunization. Consistent with the results observed in ICR (CD-1) mice, FM-SNAP induced the most potent protective IgG responses against each peptide, favoring a Th1-biased Th1/Th2 immunity, surpassing F-SNAP, M-SNAP, and MP12 vaccines. Notably, across all peptide-SNAP immunized mice, a reduction in fungal burdens and dissemination was observed within 14 days post-challenge compared to adjuvant or vehicle controls.

Our prior study demonstrated that Fba-Met6 conjugate MP12 in Adjuplex protects both immunocompetent C57BL/6 and IL-17RA k/o mice from the lethal *C. albicans* challenge, and the balanced IgG2a/IgG1 ratio played a key role (16). In this study, using highly susceptible A/J mice with different MHC haplotypes, SNAP vaccines induced higher levels of specific IgGs compared to MP12. Furthermore, SNAP vaccines demonstrated superior protection against both *C. auris* and *C. albicans* compared to MP12, as evidenced by higher survival rates and reduced fungal burden. Similarly, the peptide-SNAP platform induced robust IgG2a and IgG1 responses with balanced but Th1-biased Th1/Th2 immunity and reduced kidney CFU significantly comparable to MP12. These findings indicate that the SNAP adjuvant platform establishes an immune environment conducive to both humoral and cellular mechanisms crucial for protection against invasive candidiasis. It would be intriguing to investigate the cellular mechanisms underlying SNAP vaccine-induced protection and to validate whether IL-17RA signaling is essential for Fba/Met6-based protection against *C. auris* in this neutrophil-recruitment deficient model. In future studies, we will persist in employing our established immunocompromised mouse models of intravenous disseminated infection, which effectively mimic human clinical scenarios. These models serve as invaluable tools for assessing the *in vivo* efficacy of peptide-SNAP vaccines, including evaluations in IL-17RA knockout mice (12, 19, 31, 35).

ELISpot assays show that immunization with peptide-SNAP induces high *in vitro* production of IL-2, IFNγ, and TNF-α upon re-stimulation with Fba. IFNγ is a hallmark cytokine of Th1 immune responses, which are crucial for combating intracellular fungal infection (36). TNFα is also produced by Th1 cells and plays a role in inflammation and immune defense. The higher levels of specific TNFα/IFNγ-producing cells suggest a robust Th1 immune response, which is beneficial for controlling invasive *Candida* infections. Furthermore, IFNγ and TNFα are both important for the activation of macrophages and other phagocytes, which are essential for eliminating fungal pathogens. Therefore, the higher levels of specific TNFα/IFNγ-producing cells suggest an enhanced effector function of immune cells involved in fungal clearance. The presence of specific IL-2-producing cells indicates the generation of memory T cells, which are crucial for long-term immunity against fungal pathogens. These memory T cells can rapidly respond upon re-exposure to the fungus, leading to a more effective and rapid immune response. IL-2 and IFNγ are cytokines associated with the activation and proliferation of T cells, particularly CD4^+^ T helper cells and CD8^+^ cytotoxic T cells. SNAP systems have previously been shown to effectively induce CD8^+^ cellular immunity with short peptides (13). Higher levels of specific IL-2- and IFNγ-producing cells indicate an enhanced T-cell response against the fungal pathogen targeted by the vaccine. On the other hand, several studies reported the importance of CD8^+^ T cells in combating *Candida* infection (37). Fba- or Met6-specific CD8^+^ T-cell responses can also be induced by the vaccine, and CMI response associated with antibodies could corroborate with the same favorable outcome in vaccinated mice.

In summary, our findings suggest that both antigen-specific antibodies and cell-mediated immunity could account for the protection conferred by liposome-displayed peptide vaccines against disseminated *C. auris* infection.

### Conclusion

There are currently no antifungal vaccines available. Peptide-based anti-*Candida* vaccine, when coupled with the SNAP platform, demonstrates notable advancements. The FM-SNAP vaccine not only elicits stronger antibody responses compared to the prior Fba-Met6 conjugate vaccine (MP12) comparator vaccine but also achieves this with significantly lower antigen doses. The SNAP vaccine platform exhibits potential for delivering multivalent protective peptide epitopes, thereby limiting the fungal pathogen’s ability to evade the immune system.

Considering the intraspecies and interspecies antigenic variations among fungi, a multi-epitope vaccine becomes appealing for providing broader protection. Such a vaccine can target multiple T-cell subsets and antibody-mediated pathways. Our findings support the idea that the SNAP adjuvant platform effectively presents conserved protective peptide epitopes on highly immunogenic mosaic nanoparticles, paving the way for a pan-*Candida* vaccine capable of generating multifaceted immune responses.
